# Optimal Homogenization of Perfusion Flows in Microfluidic Bio-Reactors: A Numerical Study

**DOI:** 10.1371/journal.pone.0014574

**Published:** 2011-01-27

**Authors:** Fridolin Okkels, Martin Dufva, Henrik Bruus

**Affiliations:** Department of Micro- and Nanotechnology, Technical University of Denmark, DTU Nanotech, Kongens Lyngby, Denmark; Dalhousie University, Canada

## Abstract

In recent years, the interest in small-scale bio-reactors has increased dramatically. To ensure homogeneous conditions within the complete area of perfused microfluidic bio-reactors, we develop a general design of a continually feed bio-reactor with uniform perfusion flow. This is achieved by introducing a specific type of perfusion inlet to the reaction area. The geometry of these inlets are found using the methods of topology optimization and shape optimization. The results are compared with two different analytic models, from which a general parametric description of the design is obtained and tested numerically. Such a parametric description will generally be beneficial for the design of a broad range of microfluidic bioreactors used for, e.g., cell culturing and analysis and in feeding bio-arrays.

## Introduction

The development of microfluidics, to handle minute amounts of fluids, is currently revolutionizing fluid transport in the field of analytic cell-biology: Traditionally, cells are cultured in so-called batch cultures in a flask and an experiment is typically initiated by adding an agent. After a certain time, such as a day or two, the response of the agent is studied using typically only one reporter such as fluorescence. In order to increase throughput, cells can, at present, be cultured and assayed in robotically controlled 96 or 384 well plates. By contrast, culturing of cells on a microfluidics device gives a range of new possibilities [1] e.g. studying cell mobility in real time when exposed to stable continuous gradients [2]. Furthermore, combinatory experiments can be performed on chip that are based on arrays of interconnecting chambers [3], [4].

The inlet design presented in this paper introduce a number of improvements to current perfused bio-reactors: The creation of uniform flow conditions all over the bio-reactor ensures homogeneous cell conditions both with regards to concentrations of externally supplied growth-factors and to the shear induced on the cells by the perfusion flow. Too small a height of cell culture chips is inhibiting cell growth [5]–[7], and in Refs. [8], [9] it has been shown that the chamber height must exceed 1.5 mm in order to provide identical culturing conditions as in traditional cell culture flask. On the other hand, to ensure laminar flow conditions, a small height is preferred. Therefore in the case of cell-culturing chips, a chamber height of 1.5 mm is optimal. In other cases, such as for micro-array hybridization chambers, the functionality indeed benefit from far smaller reactor-channel heights, where volume needs to be minimized in combination with a maximization of reaction area.

In recent years different bio-reactors have been constructed, where the uniformity of the perfusion flow along the reaction area has been achieved at the expense of a large hydraulic resistance across the whole bio-reactor [3]. One example is the Micro cell-culture chamber by M. Stangegaard et al. [8], where the fluid is directed from a wide reservoir through a large number of small parallel channels. This barrier creates a large pressure drop which give rise to the uniform flow. From the inlet structure described from this work, the same uniform flow-field can be achieved with a significantly lower pressure-drop. This opens up the possibilities of driving the perfusion flow by low-power methods such as e.g. buoyancy force, which recently has been used to drive other microfluidic devices [10].

Our novel design also reduces the fluid volume used in creating the uniform flow, which is crucial both when dealing with expensive biochemical samples or to avoid dilution of small samples. Additionally it will enable a better analysis of fast cell-reaction kinetics with high time resolution.

The paper is organized as follows: In section “Layout” the general bio-reactor layout is outlined together with an introduction of the related characteristic parameters. In section “Optimization” the optimal structure of the perfusion inlet is found, first by the general method of topology optimization, which imposes no constraints on the topology of the structure. The resulting structure is further refined by the method of shape optimization. The optimized geometry is in section “Comparison with alternative expansion geometries” compared to two simple expansion design, while in section “Design guide” the results are summarized in a design guide. The analysis and the design guide is further verified by full 3D simulations in section “Direct 3D simulation”. Finally a conclusion is given in the end.

## Methods

**Figure 1:**
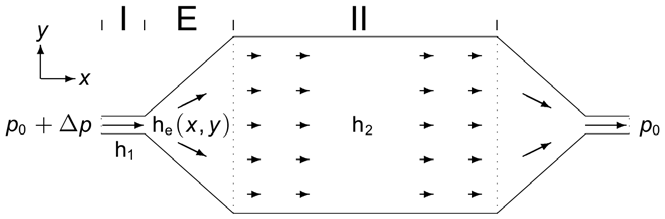
A sketch of the bio-reactor with its three sections. (I) the perfusion inlet of constant height 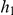, (E) the expansion chamber of spatially varying height 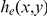, and (II) the main reactor area of constant height 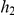. The perfusion flow, driven by the pressure drop 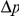, is indicated by arrows.

The generic microfluidic bio-reactor layout used in this work is illustrated in Figure 1. It consists of a single microchannel perfusion inlet (I) of constant height 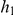, which broaden out in an expansion chamber (E) of varying height 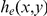 to distribute the fluid over the much wider and more shallow main reactor (II) of constant height 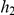, where the cells are immobilized. All vertical channel and chamberheights in the 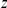 direction are much smaller than any lateral length scale in the 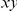 plane; the bio-reactor is thus flat.

The main objective is to obtain a uniform flow in the main reactor with minimal pressure drop 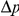 and with a minimal volume of the expansion chamber. This is achieved by carefully designing variations in chamber height 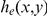 of the expansion chamber. As the constant inlet channel height 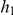 is assumed larger than the constant height of the main reactor area 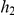, the height variation in the expansion chamber (E) will be bounded by these two heights:
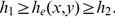
(1)The whole bio-reactor is assumed symmetric both through a central vertical and a central horizontal axis, and as a consequence only the upper left part will be dealt with here.

As we consider only low concentrations of the solutes and a constant temperature, the density 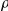 and viscosity 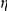 of the buffer liquid are constant in space, and the flow is determined by the geometry of the reactor and the applied pressure drop 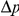 driving the flow. As a consequence of the assumed flatness of the bio-reactor, the pressure 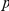 does not vary in the vertical 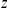 direction, i.e. 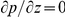. Moreover, due to the small heights, viscous damping from the top and bottom plates of the bio-reactor dominates the fluid flow and makes the flow laminar. This is evident from the value of the Reynolds number 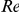 given the low flow velocities, 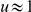 mm/s, and small length-scales, 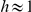 mm of the system: 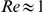.

In this flow regime it is useful to work with the 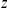-averaged 2D velocity field
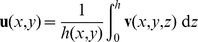
(2)of the full 3D field 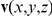. To a good approximation 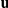 fulfills the 2D Brinkman-Darcy equation [11],
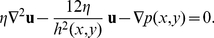
(3)Here the prefactor 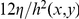, also denoted the damping coefficient 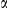,
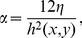
(4)is reminiscent of the 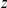-part of the Laplace operator in the full 3D-description, and it represents the dominant part of the viscous damping of the liquid in the system.

For possible continuous changes in the height 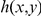 of the expansion region (E), the 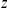-averaged 2D velocity field is not divergence-free due to mass-conservation, but an additional tern arises: 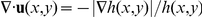. In the case of possible discontinuous jumps in height along an interface, this correction becomes the following new boundary condition on the interface:
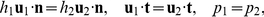
(5)where the height-subscript is extended to the corresponding velocities and pressures.

**Figure 2:**
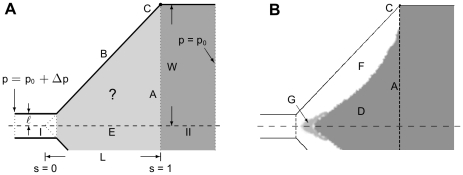
The expansion champer. (a) The geometry of the expansion chamber E. The parameters related to geometry and flow are defined together with specific segments and points used for later reference. The optimal transition between the inlet height 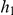 (white area) and the main reactor channel height 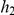 (gray) occurs inside the expansion region E (light gray). (b) Topology optimized height variation in the expansion region. The height changes almost step-like from the white region F (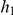) to the gray region D (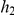). Note the small region of intermediate height marked G.

Working with the 2D-restricted description, the detailed geometry of the expansion chamber is illustrated in Figure 2. The important in-plane length scales are the length 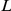 of the expansion chamber, the width 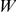 of the main reactor, and the width 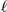 of the inlet. To achieve a uniform flow in the main chamber, the pressure along the line A dividing the expansion chamber and the main chamber must by constant. Consequently, the spatial variation of the expansion chamber height 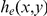 must be optimized in order to get as homogeneous pressure along line A as possible.

### Optimization

To enable optimization of the system two additional parts are introduced. First, a set of design variables 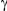, which uniquely characterizes all available configurations in the optimization problem, and for which a unique solution 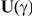 to the system exists. Second, an objective function 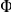 which quantifies how well a given configuration of the system performs. By convention 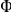 has to be minimized in order to achieve the optimal solution, and generally the objective function can depend on the design variables and the related solution of the system 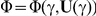.

As alluded to in the previous section, we base our objective function on the homogeneity of the pressure along the cross-section A, since a uniform pressure there will lead to the required uniform flow field in the main reactor. In the following, this objective will be expressed in two different ways, depending on the given optimization methods.

#### Topology optimization of the spatial height variation

To search for the globally optimal solution, and not a priori exclude any non-intuitive solutions, we will not rely on any pre-described variation of the height. Therefore, we begin by applying the method topology optimization [12], which by definition is independent of the topology and therefore unlimited in its search for the optimal bio-reactor design. The method of topology optimization was first applied to the field of structural mechanics [13], and have been recently implemented to the field of microfluidic systems [14], [15] and chemical microreactors [16].

Arbitrary height variations of 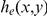 can be realized by representing the height as a variation of the design variable field 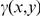, where 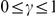. To cover the range of heights defined in equation (1), the design variable is assigned the value 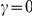 to describe chamber heights equal to the inlet height 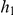 and the value 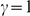 for heights equal to the main reactor height 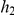. In the expansion chamber, now denoted the design region 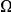, the design field can take any value 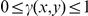 to describe all possible height variations 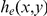.

The actual implementation, method and procedure of topology optimization will not be touched upon here, as it is fully described in the work of Olesen, Okkels and Bruus [15]. Still what is essential for this work is the objective function 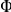, which has to be chosen with care. To obtain a numerically stable search we define 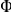 as the square deviation of the pressure around a reference pressure 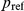 along A:
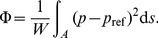
(6)We choose 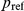 as the pressure at the far corner C in the case where the expansion chamber has the same height 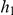 everywhere as the inlet.

Figure 2(b) shows the resulting optimal height distribution 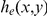 for the following set of parameters: 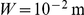, 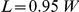, 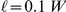, 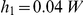, and 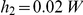, where the gray-scale color-coding spans from height 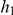 in white down to 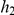 in gray. We define the ratio 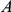 between the two damping coefficients 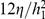 and 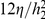 as
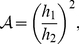
(7)and get the value of 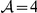 for Figure 2(b).

As mentioned earlier, any change in height produces a correction-term to the continuity equation, when using a 2D-restricted description. It turns out that both ways of implementing this correction in the topology optimization problem fails, due to the very nature of the method. First, the free variations of the design field in topology optimization prohibits any interface to be defined a priori, and therefore the boundary conditions of equation (5) cannot be applied in this step of the optimization procedure. Second, it turns out that the solutions of the topology optimization problem involves sharp transitions in the height, limited by the grid-meshing length-scale of the finite element method. Therefore when including the correction-tern to the continuity equation, a fluid source is added to single mesh-elements, and this destabilizes the convergence of the method. The way to work about this limitation, is to add the boundary conditions of equation (5) to the shape-optimization method, applied later in the optimization process, after the shape-optimization has been preliminarily compared to the topology-optimization.

From the topology optimized solution in Figure 2(b) we see that among all possible height variations, the optimal design consists of a single sharp transition between a region of inlet height 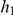, and a region of main reactor height 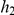. Only very close to the inlet channel is seen an ambiguity which indicate the possible existence of a region of intermediate height. From topology optimizations for other parameter-values, similar solutions arise with a sharp transition between regions of height 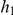 and 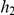, and consequently we conclude that such a single-connected transition is indeed the optimal solutions of the problem.

**Figure 3:**
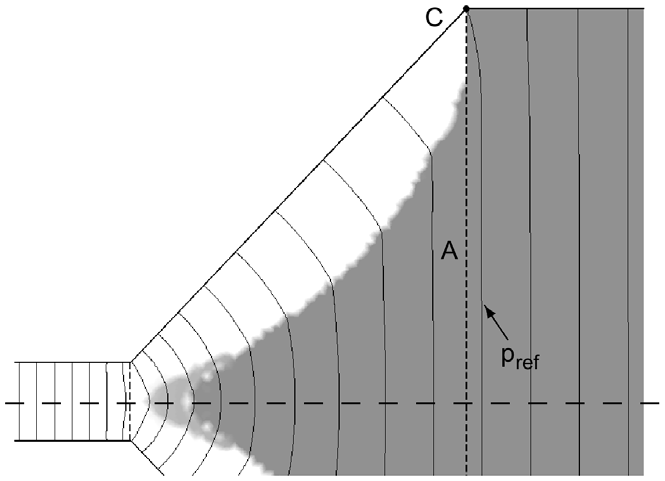
The contour lines of the pressure 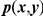 in the topology optimized design. The contour 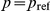, going through the corner C is marked.

To evaluate the quality of the topology optimized solution, we plot in Figure 3 the pressure contour lines of the solution in Figure 2(b), including a contour line corresponding to the value 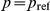 which goes through the corner 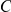 of the expansion chamber. Except close to the upper side wall, the pressure is seen to be uniform at the entrance of the main reactor, and it decreases uniformly throughout the whole extension of the main reactor.

The chosen parameters used for the solution in Figures 2(b) and 3 represent a rather extreme case, i.e., a combination of a small height-difference 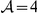 and a wide expansion 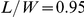. As a result the transition extends nearly through the whole expansion region, but for all common parameters, the type of solution remains optimal.

From the results of the topology optimization it is therefore natural to proceed with the shape optimization method, which compared to topology optimization involves fewer design parameters, is faster, and is numerically more stable.

#### Shape-optimization

**Figure 4:**
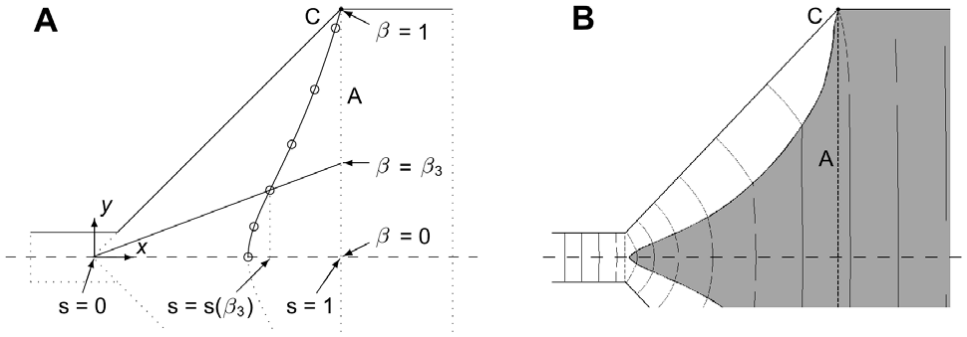
The shape-optimization problem. (a) Setup for the parametrization. Variables 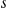 and 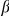 parameterize the normalized 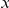 and 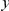-axis respectively, and the control-points 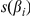 are shown by circles with the interpolated interface curve in solid. (b) The pressure contours (thin lines) of the shape optimized positioning of the interface line 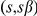 (thick line). Similarly to Figure 3 the pressure contour are originating from the upper corner C.

In shape optimization the interface line between the heights 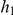 and 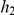 in the transition chamber is given by a cubic interpolation line through a number of control points 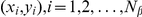 as shown in Figure 4(a) with 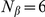. It is convenient to parameterize the points by the expression
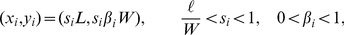
(8)which ensures that all points lie within the expansion chamber. By fixing the factors 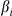 by 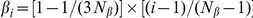, a relatively even distribution of the control points is also ensured. During the optimization process the position of the interface line is changed by adjusting the control points 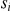.

The optimization is carried out by a simplex-method relying only on values of the objective function 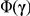, and not its partial derivatives 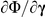. To ensure efficient convergence of the given simplex method, it is beneficial to assign initial values around unity for the design variables 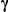. Furthermore, the method is unbound i.e. the design variables must give rise to a well-defined geometry regardless of their value. All this is accomplished by using the arcus tangent function:
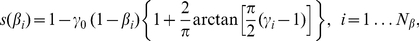
(9)with
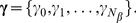
(10)We use 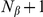 design variables to determine 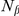 shape parameters because a faster convergence is achieved by adjusting the extend of the whole interface by a single parameter 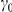. Furthermore, equation (9) let the initial configuration of
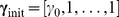
(11)give rise to a well-defined, straight interface line reaching from the position 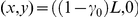 to the upper corner.

Now that the interface by definition extends to the upper corner, this constraint does not need to be included in the objective function 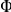 of the shape-optimization, and 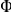 can therefore be defined with the sole purpose of achieving a uniform pressure along segment A:
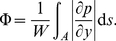
(12)


The actual optimization of the design-variables follows two steps: First a rough initial interface is found by using the initial setup of equation (11), and only adjusting the single variable 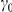, using a simple Matlab implementation of a bounded golden section search with combined parabolic interpolation [17]. Once a suitable straight initial interface is found, the actual shape is obtained using a direct unbounded simplex search method, also implemented in Matlab [18].

## Results

First, the shape-optimization method has to be validated with respect to the topology optimized solution, shown in Figures 2(b) and 3. The same parameter-values were used, and the resulting shape-optimization shown in Figure 4(b), is indeed similar to Figure 3. When comparing the results of the two optimization methods, it is observed that both the shape of the interface and the corner-pressure contour matches very well. Thereby we conclude that the shape-optimization is appropriate for the further analysis of the optimized interface.

Again it should be noted that the set of parameters used in Figures 2(b), 3, and 4(b) is an extreme case, and therefore the shape-optimization method has been further tested to ensure the validity of this simple type of solutions.

### Comparison with alternative expansion geometries

**Figure 5:**
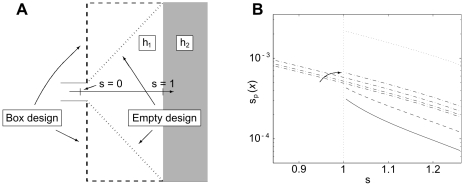
A study of alternative expansion geometries. (a) The expansion region in the *Empty design* is completely filled with inlet height 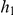, while the *Box design* has a simple box of height 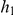 as expansion region. (b) Plot of the standard deviation 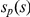 of the pressure vertically across the expansion region as a function of the normalized horizontal position 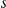 into the region, as seen in (a). The different curves are topology optimized (solid line), shape optimized (dashed line), empty design (dotted line), and the four types of box design (dot-dashed lines), where the arrow marks the increasing order of the following values 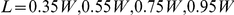.

Now that the design of the expansion region has been optimized, it is natural to compare its efficiency to other alternative expansion designs. The first obvious candidate is to uniformly fill the existing expansion region with height 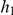 i.e. to remove the topology optimization distribution of height 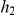, and this we will call the *empty design*. The next design comes as we replace the expansion region with a simple box of width 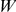 and height 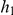, and this will be called the *box design*. Both alternative designs are shown in Figure 5(a). To compare these new candidate designs to the optimized designs, we will measure the homogeneity of the pressure around the end of the expansion region. To get an quantitative measure of the homogeneity of the pressure in the first part of the reactor, we measure the standard deviation 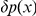 of pressure across the width along the 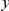-axis of the reactor part for a fixed 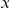-coordinate:
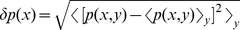
(13)where 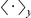 is the mean along the 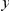 direction. Generally 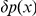 will decrease exponentially with the distance into the uniform reactor-part, and therefore 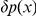 will appear as an approximately straight line when shown shown in a log-linear plot as a function of 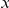, see Figure 5(b).

From the measurements presented in Figure 5(b) it is first noted that both optimized designs produce a more homogeneous pressure-field than the alternative designs. While the empty design give the poorest results, the box design comes closer to the shape optimized design, and this tendency strengthen when moving the interface closer to the inlet e.g. for 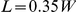. Since the box design evens out the pressure due to the translation invariant properties in the reactor part, there is a limit in how fast this can happen, as reflected in the slope of the dash-dotted lines in Figure 5(b). On the contrary, the optimized designs aims at homogenizing the pressure by designing the expansion-parts, and therefore their corresponding slopes are steeper than the box design. As a result, the optimized designs are most efficient in quickly producing homogeneous pressure-fields.

The hydraulic resistance 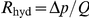 of the expansion-regions for all the presented designs are in the range 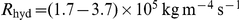, which is five orders of magnitude smaller than the numerically estimated 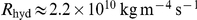 for the micro cell culture [8].

The optimized designs possesses another advantage, since the fluid-volume of the corresponding expansions regions are significantly smaller than any of the other designs mentioned. The fluid-volume of the different expansion-regions has been calculated/measured and is presented in Table 1. Also presented in the table is the volumes relative to the Shape optimized design, and this clearly shows that extra fluid-volume is significantly higher especially for the box designs.

**Table 1:** The volume of the different designs of the expansion chamber.

Type	TO	SO	ED	BD-0.95	BD-0.75	BD-0.55	BD-0.35
Vol ( 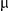 L)	27.7	27.6	37.6	68.4	62.0	55.6	49.2
Vol/Vol(SO)	1.01	1	1.36	2.48	2.25	2.01	1.78

Abbreviations used: TO = Topology Optimized, SO = Shape Optimized, ED = Empty Design, and BD-X = Box Design, with the corresponding length-to-width fraction 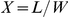. Second row shows the volumes in relation to the Shape optimized design.

From the above results we conclude that the optimized designs are generally better than the alternative designs, and we will therefore in the following present a general description based on a vast range of different shape optimized designs.

Knowing now the basic shape of the height interface in the expansion region, we can now apply the right mass-conserving boundary conditions of equation (5) to the interface, and thereby improve the model upon which the following design guide is based.

### Design guide

It is possible to match the numerically optimized geometry by simple theoretical models, which only depend significantly on the parameters 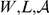, as the remaining parameters 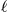 and 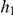 only introduce minor corrections. By fitting the resulting interface obtained in these models for given parameters to the corresponding shaped optimized interface, we obtain an approximate parametrization which can serve as an easily applicable guide for practical design purposes.

**Figure 6:**
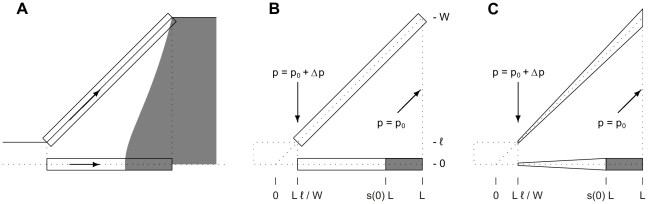
The concept behind the simple analytic models. (a) A comparison between the flow along two distinct flow stream in the expansion chamber. (b) The plug flow model, and (c) the radial flow model. Also shown are the involved parameters and constraints. The gray area corresponds to regions of height 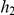.

The basic idea behind the simple models is sketched in Figure 6. Given the laminar nature of the flow, we consider an idealized narrow flow stream stretching from the inlet, across the expansion chamber, to the entrance of the main reactor. The first part of the stream, which is the lower part of Figure 6a, goes along the horizontal symmetry-axis and starts at the inlet where the hydraulic damping factor is 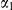 given by the height 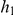, see equation (4). Then, at the point 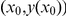 the stream hits the interface, and continues horizontally to the point 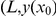 with hydraulic damping factor 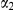. Along all streams, the hydraulic resistance is proportional to the effective length 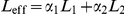, where 
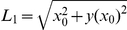
 and 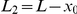 is the length of the first and second part of the stream, respectively. Since we seek the shape 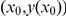 of the interface giving rise to the same pressure drop along the streamlines, all streamlines must have the same effective length. The specific form of the effective length, with its squares of 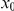 and 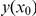, then leads us to expect an expression for 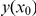, or in dimensionless form, an expression for 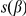 of the form
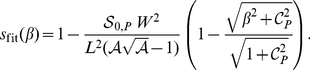
(14)


The proposed expression for 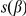 is of course not exact, but by calculating the shape optimized interface for a large number of parameter values, we can fit equation (14) and make an statistical analysis of the obtained fitting parameters 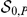 and 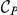.

The resulting explicit parametrization is the main result of the article, and becomes
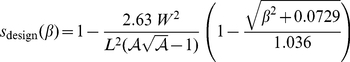
(15)To summarize, equation 15 above explicitly gives the dependence of 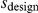 as a function of 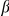, that provides a parametric description of the 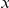,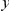 coordinates of a curve (see equation 8) defining the transition interface in the expansion region between the inlet height 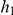 and the main reactor channel height 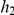. Figure 4a illustrates how 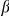 is the normalized vertical position and 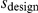 is the related horizontal position of the interface within the expansion region.

**Figure 7:**
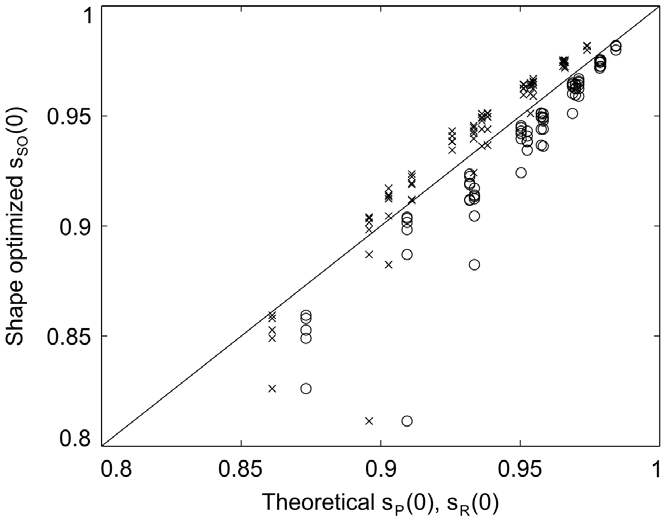
Model predictions vs. optimized shapes. A comparison of the calculated beginning 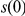 of the transition region at the center axis between the simple model predictions and the shape optimization model. The comparison includes the simple model (crosses) and the radial model (circles), and a perfect match would lie at the diagonal (solid line).

The parametrization, given in equation 15, is deduced for a plug flow model, which is shown in Figure 6b, while a more refined radial model, seen in Figure 6c, can only be solved numerically. A comparison between the two models, is seen in Figure 7. Here, the position 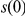 of the interface at the center axis in the simple models for a large number of parameter values is compared to that of the shape optimized model. These results show no improvement by the radial model, and therefore it is adequate to base the design guide of equation 15 on the plug model.

The design guide does generally a very good job, but it maybe worth adjusting the parameters used above (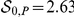, and 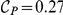) if the flow-homogeneity is very crucial. The best results are obtained within the following range of parameters: 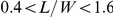, 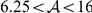, 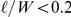, and 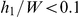. This range should be met naturally for most applications, and since we have based this work on creeping flow, the Reynolds number of the perfusion flow should be kept below or around unity.

In all, we thus find that equation 15 can serve as a fairly accurate design guide, applicable for designing microfluidic bio-reactors.

### Direct 3D simulation

**Figure 8:**
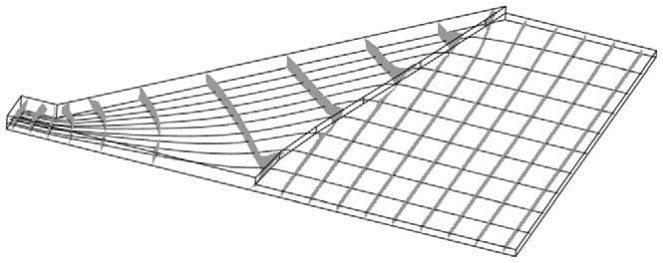
Direct 3D numerical solution of an optimized design. Following the parametrization guide of equation 15. Pressure iso-surfaces are gray, and streamlines are solid lines. Parameters are 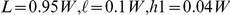, and 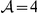.

Up to this point we have relied on the 2D flow model based on the Brinkman-Darcy equation. To validate this approach and test the guideline parametrization of equation (15), we made a full 3D direct numerical simulations of the derived bio-reactor design for a given set of parameters. The resulting system was modeled and solved in Comsol using a ordinary laptop computer, and the solution is presented in Figure 8, where both iso-surfaces of the pressure and streamlines are showed inside the computational domain.

Similar to the earlier quasi 3D solutions of the optimized design, the pressure is nicely homogenized in the region of main reactor height, and also the streamlines arrange parallel through the main reactor. We take these results as a clear validation of the lubrication approach used in this work. Besides, the homogeneous flow produced by the design in Figure 8 emphasizes the value of derived design and the parameterizations guide of equation (15).

## Discussion

To increase the utilization of continually feed microfluidic bio-reactors, we have optimized the flow-geometry of the reactor as to expose all immobilized organisms or substances to a very homogeneous flow field. From this we have derived a general guide-line of how to construct the optimal design for a broad range of reactor-dimensions.

As the overall height of the system is much smaller than the remaining physical dimensions, the 3D fluid flow can essentially be described as a 2D fluid flow using a lubrication theory approach, where an additional volume-force arise from the viscous drag by the upper and lower channel-walls.

In this work we first achieved an optimal flow-geometry by applying the free-form method of topology optimization. As the resulting shape in the design had a simple single-connected topology, we subsequently applied shape-optimization to obtain the different optimal geometries for various reactor-dimensions. From this analysis, we have constructed a general parametrization of an optimal design, which has been validated by direct 3D simulations.

The design produces the homogeneous flow with a very low pressure drop, and this will dramatically reduce the power needed to drive the perfusion flow through the system. This opens the possibilities of driving the perfusion in radically new ways e.g. by buoyancy effects. Furthermore the fluid-volume of the flow-homogenizing design is minimized, which is essential when dealing with very limited fluid-samples.

Besides applying the design to bio-reactors, it is also applicable to many other microfluidics system requiring perfusion of a large squared area, such as DNA and protein microarrays and investigation of tissue slices using fluorescent in situ hybridization or immuno chemistry, where samples typically are limited.
